# Sex differences in comorbidities and mortality risk among patients with chronic obstructive pulmonary disease: a study based on NHANES data

**DOI:** 10.1186/s12890-023-02771-3

**Published:** 2023-11-29

**Authors:** Na Li, Xiaoli Li, Minjie Liu, Yakang Wang, Junning Wang

**Affiliations:** 1 Department of Respiratory, Honghui Hospital, Xi’an Jiaotong University, Xi’an, Shaanxi 710054 People’s Republic of China; 2 Department of Orthopaedics, Honghui Hospital, Xi’an Jiaotong University, Xi’an, Shaanxi 710054 People’s Republic of China

**Keywords:** Comorbidities, COPD, Sex differences, Survival

## Abstract

**Background:**

Patients with chronic obstructive pulmonary disease (COPD) commonly have coexisting comorbidities that contribute to higher exacerbation frequency, poorer health status, and increased all-cause mortality; however, there are only a few studies available on the sex discrepancy in the comorbidity distribution and outcomes among COPD patients, and there is limited information about the discrepancy in all-cause mortality between men and women.

**Methods:**

Based on data from the U.S. National Health and Nutrition Examination Survey conducted between 2007 and 2012, we compared participants aged 40–79 years with spirometry-defined COPD to compare the prevalence of comorbidities between men and women. The survival of the subjects was documented, and the sex discrepancy was determined using Kaplan–Meier analysis. Comorbidities and all-cause mortality were analyzed by using a Cox proportional hazards model to determine their strength of association in different sex groups.

**Results:**

Compared to men, women had a significantly higher prevalence of asthma (OR 1.93, 95% CI 1.46 to 2.57, p < 0.001) and arthritis (OR 1.77, 95% CI 1.39 to 2.24, p < 0.001). Women had a significantly lower prevalence of coronary heart disease (OR 0.48, 95% CI 0.27 to 0.87, p = 0.015) and gout (OR 0.42, 95% CI 0.25 to 0.67, p = 0.001). Kaplan–Meier analysis revealed that compared with that of the female group, the survival rate of the male group was significantly lower (p < 0.001). Among men, the presence of anemia (HR 2.38, [95% CI 1.52–3.73], p < 0.001), gout (HR 1.55, [95% CI 1.04–2.30], p = 0.029) and congestive heart failure comorbidities (HR 1.85, [95% CI 1.12–3.04] p = 0.016) was associated with a higher risk of mortality; among women, the presence of anemia (HR 2.21, [95% CI 1.17–4.20], p = 0.015) and stroke (HR 2.04, [95% CI 1.07–3.88], p = 0.031) comorbidities was associated with a higher risk of mortality after adjusting for age, race/Hispanic status, BMI, smoking status, FEV1% predicted and prevalent comorbidities.

**Conclusions:**

COPD-related comorbidities and all-cause mortality were discrepant between men and women, and men had poorer survival than women in the nationally representative data that were analyzed.

## Background

Chronic obstructive pulmonary disease (COPD) is a chronic respiratory condition that can be prevented and treated and is characterized by persistent respiratory symptoms accompanied by airflow limitations that are not fully reversible [1]. COPD is a major global cause of morbidity and mortality, is projected to become the seventh leading cause of the disease burden and the third leading cause of death worldwide by 2030 and is currently considered a major public health issue [2, 3]. Patients with COPD commonly have coexisting comorbidities, including diabetes mellitus (DM), cardiovascular disease (CVD), hypertension, osteoporosis, anemia, metabolic syndrome, sleep disorders, dyslipidemia and several types of cancer or malignancy [4, 5]. Additionally, in COPD patients, these comorbidities can contribute to higher exacerbation frequency, higher levels of polypharmacy, an increased number of unscheduled hospital admissions, poorer health status, and increased all-cause mortality, resulting in an increased disease burden for people with COPD [6, 7]. Therefore, recent COPD guidelines emphasize the management of comorbid conditions [8]. An increasing amount of evidence suggests that sex contributes to differences in presentation, severity and outcomes among patients with COPD [9]. However, there are only a few studies available on sex differences in the comorbidity distribution and outcomes among COPD patients, and information about sex differences in all-cause mortality is limited. This study evaluated the prevalence of different comorbidities among participants with COPD by sex and determined the difference in survival between males and females with COPD in a large nationally representative dataset in the United States. Assessing sex differences in COPD-related comorbidities might assist us in developing better management strategies, thereby contributing to a reduced impact and a substantial improvement in patient quality of life [10].

## Methods

### Data sources

Data from the 2007–2008, 2009–2010 and 2011–2012 cycles of the National Health and Nutrition Examination Survey (NHANES) were analyzlysed for this study because these survey cycles included spirometry testing data. NHANES is a cross-sectional, noninterventional survey, and a multistage complex cluster sampling design is used to provide nationally representative data on the civilian population of the United States; the study is conducted by the Centers for Disease Control and Prevention (CDC). A household interview and a standardized physical examination at a mobile examination centre (MEC) laboratory were used to collect the data. We linked data from National United States Death Index files updated to 31 December 2019 to record mortality status and follow-up time for all subjects. The survey participants’ survival time was defined as the time from the date of the NHANES interview to the date of mortality from any cause or the end of follow-up (before December 31, 2019). All data are available and free to download at https://www.cdc.gov/nchs/nhanes/ index.htm and https://www.cdc.gov/nchs/data-linkage/mortality.htm. The present study used a public database provided by NHANES, so approval by the Institutional Ethical Review Board was not needed.

### Participants

**Fig. 1:**
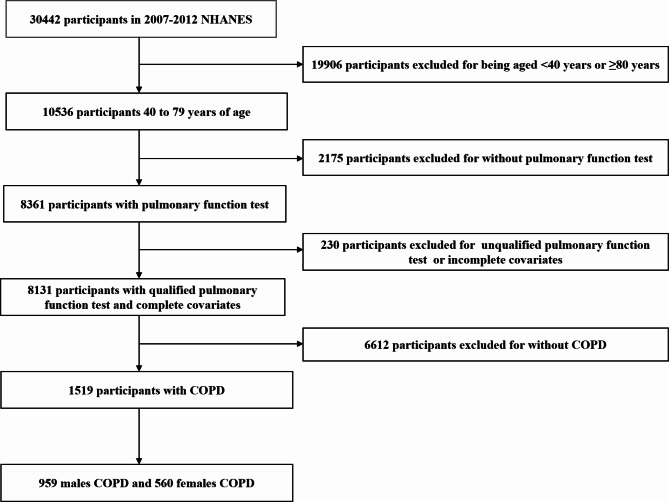
Flowchart of study participant selection process Abbreviations: NHANES National Health and Nutrition Examination Survey, COPD chronic obstructive pulmonary disease

During 2007–2012, among the 30,442 subjects in 3 cycles, 10,536 subjects were 40 years or older and younger than 79 years. A total of 8361 subjects were invited to participate in the spirometry test. Among them, 8131 participants had qualified pulmonary function tests and complete covariate information. A total of 230 participants were excluded from spirometry due to unqualified pulmonary function tests or incomplete covariate information. An additional 6612 participants were excluded for not having COPD. In total, 1519 participants (959 men and 560 women) with COPD were eligible for further analysis. Participants’ detailed screening procedures are shown in Fig. 1.

### Spirometry test and COPD

During NHANES 2007–2012, subjects who met certain inclusion criteria were offered spirometry testing. FEV1 and FVC absolute values were taken directly from the database; then, calculation of FEV1% predicted (FEV1% pred) and FVC percent predicted (FVC % pred) values was performed using the reference equation considering race, sex, age and height based on an analysis of the general population in the United States NHANES [11]. The analysis was restricted to males and females between the ages of 40 and 79 with COPD defined by spirometry [prebronchodilator forced expiratory volume in one second (FEV1)/forced vital capacity (FVC) < 70%]. COPD severity of obstruction based on the FEV1% predicted value was categorized as severe-to-very severe (FEV1 < 50% predicted), moderate (50% ≤ FEV1 < 80% predicted), and mild (FEV1 ≥ 80% predicted) according to classification guidelines developed by the Global Initiative for Chronic Obstructive Lung Disease (GOLD) [8].

### Definition of comorbidities

Comorbidities (asthma, arthritis, gout, angina pectoris, coronary heart disease, congestive heart failure, heart attack, cancer or malignancy) were defined as a self-reported physician diagnosis. In addition to this, as hypertension, self-reported antihypertensive medication use, systolic blood pressure (SBP) ≥ 140 mmHg, or diastolic blood pressure (DBP) ≥ 90 mmHg was considered [12], an average of 1 to 3 blood pressure measurements was used to calculate SBP and DBP. Diabetes mellitus (DM) was defined by a fasting plasma glucose level ≥ 126 mg/dL, a percentage of total haemoglobin ≥ 6.5%, the use of insulin injections, or the use of diabetes pills to lower blood sugar [13]. Dyslipidaemia was defined as the use of lipid-lowering medications, a low-density lipoprotein cholesterol level > 130 mg/dL, a high-density lipoprotein cholesterol level < 40 mg/dL, or a triglyceride level > 200 mg/dL [14]. In men, anemia was defined as a hemoglobin level lower than 13 g/dl, and in women, anemia was defined as a hemoglobin level lower than 12 g/dl [15]. Underweight was defined as a body mass index (BMI) < 18.5 kg/m^2^, normal weight was defined as 18.5 kg/m^2^ ≤ BMI < 25 kg/m^2^, overweight was defined as 25 kg/m^2^ ≤ BMI < 30 kg/m^2^ and obesity was defined as a BMI ≥ 30 kg/m^2^ according to the CDC guidelines [16]. By self-reported activity, smoking status was classified as never smoking or ever smoking based on whether the participant smoked at least 100 cigarettes in his or her lifetime. According to their smoking status at the time of the interview, ever smokers were further classified as current or former smokers. Age was categorized as 40–64 and 65–79 years.

### Statistical analysis

Statistical analysis was performed using Stata version 16 (College Station, Texas, US) and R version 4.2.1 (R Foundation for Statistical Computing, Vienna, Austria). The weights of the sample were used to account for the different selection probabilities and the complex NHANES sample design. Survey sample weights were used in all the analyses. We used Student’s t test for continuous variables and the chi-square test for categorical variables to compare differences in baseline data between men with COPD and women with COPD. For any comorbidity, we calculated the prevalence and 95% confidence interval (CI) by male and female groups that were representative of the noninstitutionalized U.S. population aged 40–79 years and used log binomial logistic regression to estimate adjusted prevalence ratios and 95% CIs comparing the female COPD group to the male COPD group, adjusting for age, race/Hispanic status, BMI, smoking status, FEV1% predicted and prevalent comorbidities (including hypertension, congestive heart failure, coronary heart disease, angina pectoris, heart attack, stroke, dyslipidemia, diabetes, gout, asthma, arthritis, anemia, cancer or malignancy and sleep disorder). We estimated the survival probability at the end of the follow-up period (by 31 December 2019) between the male COPD patients and female COPD patients using a Kaplan–Meier analysis and Cox proportional hazards regression model after adjusting for age, race/Hispanic status, BMI, smoking status, FEV1% predicted and prevalent comorbidities (including hypertension, congestive heart failure, coronary heart disease, angina pectoris, heart attack, stroke, dyslipidemia, diabetes, gout, asthma, arthritis, anemia, cancer or malignancy and sleep disorder). Comorbidities and all-cause mortality were analyzed in different sex groups by a multivariable Cox proportional hazards regression model to determine their strength of association after adjusting for age, race/Hispanic ethnicity, BMI, smoking status, FEV1% predicted and prevalent comorbidities (including hypertension, congestive heart failure, coronary heart disease, angina pectoris, heart attack, stroke, dyslipidemia, diabetes, gout, asthma, arthritis, anemia, cancer or malignancy and sleep disorder). We calculated hazard ratios (HRs) with 95% confidence intervals (CIs). All statistical tests were two-sided, and a P value of less than 0.05 indicated statistical significance for all tests conducted.

## Results

### Participant characteristics

A total of 1519 patients who met the inclusion criteria were enrolled in this study, including 959 male COPD patients and 560 female COPD patients. The average ages of the male and female groups were similar, and there was no difference between the two groups in the age distributions of those aged 40–64 years and 65–79 years. There were no significant differences in racial distribution. BMI was on average higher for men (28.2 kg/m^2^) than for women (27.0 kg/m^2^). Women were more likely to be underweight (3.8%) or normal weight (39.8%); however, men were more likely to be overweight (41.7%) or obese (29.4%). Women were more likely to be never smokers (32.9%), and men were more likely to be former or current smokers (41.2% and 34.1%, respectively). Female COPD subjects had an average lower FVC% predicted (94.8% vs. 98.2%, *p* = 0.0261) and lower FEV1% predicted (78.0% vs. 82.0%, *p* = 0.0105) than men. Regarding COPD severity, women had a higher proportion of severe-to-very severe COPD (7.6%), and men had a higher proportion of mild (FEV1 ≥ 80% predicted) COPD (56.9%). Patient characteristics for both groups are presented in Table 1.

**Table 1:** Characteristics of participants with COPD aged 40–79 by sex, U.S. NHANES 2007–2012

Demographic characteristic	Men *n* = 959	Women *n* = 560	*p* value
Age (mean, SE)	62.2(0.5)	61.7(0.5)	0.9249
Age group (years)			0.3987
40–64 (%)	67.7 (63.9, 71.3)	65.4 (60.9, 69.6)	
65–79 (%)	32.3 (28.7, 36.1)	34.6 (30.4, 39.1)	
Race/ethnicity (%)			0.0648
Mexican-American	2.7 (1.8, 4.1)	1.2 (0.7, 2.0)	
Other Hispanic	2.2 (1.4, 3.4)	1.7 (0.9, 3.0)	
Non-Hispanic White	83.6 (79.2, 87.2)	84.5 (80.1, 88.1)	
Non-Hispanic Black	7.8 (6.0, 10.1	7.5 (5.6, 9.9)	
Other	3.8 (2.3, 6.2)	5.1 (2.9, 8.8)	
BMI (kg/m2) (mean, SE)	28.2(0.2)	27.0 (0.3)	0.0032
BMI category (%)			< 0.0001
Underweight	0.8 (0.4, 1.7)	3.8 (2.3, 6.1)	
Normal	28.3 (24.2, 32.7)	39.8 (34.6, 45.2)	
Overweight	41.7 (37.7, 45.8)	31.1 (26.3, 36.3)	
Obese	29.2 (25.9, 32.8)	25.4 (21.1, 30.2)	
Smoking (%)			0.0404
Never	24.7 (20.5, 29.4)	32.9 (27.5, 38.9	
Former	41.2 (37.7, 44.8)	33.5 (26.6, 41.2)	
Current	34.1 (30.0, 38.4)	33.5 (28.2, 39.3)	
Spirometry			
FVC % predicted (mean, SE)	98.2(0.8)	94.8(1.1)	0.0261
FEV1% predicted (mean, SE)	82.0(0.9)	78.0(1.1)	0.0105
GOLD categories (%)			0.0782
Mild (FEV1 ≥ 80%)	56.9 (51.0 ,62.6)	47.3 (41.3, 53.4)	
Moderate (FEV1 50–79%)	37.9 (32.8 ,43.3)	45.1 (39.0, 51.4)	
Severe-to-very severe (FEV1 < 50%)	5.2 (3.3 ,8.0)	7.6 (5.2, 11.1)	

Values are presented as weighted means (standard errors of the means) or weighted percentages (95% confidence intervals of percentages)

### Prevalence comparison

The highest prevalence of comorbidities among male and female COPD patients was for dyslipidemia (77.1% and 74.0%), and there was an equal distribution in both groups. Men had a higher prevalence of coronary heart disease (7.4%), heart attack (6.5%), and gout (7.7%). In contrast, women had a higher prevalence of asthma (25.6%) and arthritis (42.6%). Hypertension, congestive heart failure, angina pectoris, stroke, diabetes, anemia, cancer or malignancy and sleep disorders were homogeneously distributed in both sex groups. Trends in comorbidity frequency and prevalence in different sex groups are presented in Table 2.

**Table 2:** Prevalence of comorbidities (95% confidence intervals) among participants with COPD aged 40–79 by sex, U.S. NHANES 2007–2012

Comorbidity	Men *n* = 959	Women *n* = 560	*P* value
Cardiovascular disease			
Hypertension	45.9 (40.9 ,51.0)	45.8 (40.5 ,51.2)	0.9749
Congestive heart failure	4.3 (3.1 ,5.9)	3.0 (2.0 ,4.5)	0.1213
Coronary heart disease	7.4 (5.5 ,9.9)	3.4 (1.9 ,6.1)	0.0141
Angina pectoris	4.0 (2.8 ,5.8)	2.6 (1.2 ,5.4)	0.2932
Heart attack	6.5 (4.8 ,8.6)	3.9 (2.5 ,6.0)	0.0240
Stroke	3.3 (2.2 ,4.8)	4.6 (3.1 ,6.7)	0.2516
Dyslipidemia	77.1 (73.6 ,80.2)	74.0 (69.0 ,78.5)	0.2190
Diabetes	12.5 (10.2 ,15.4)	9.3 (7.2 ,12.0)	0.0661
Gout	7.7 (5.5 ,10.7)	3.5 (1.9 ,6.4)	0.0088
Asthma	16.2 (12.7 ,20.4)	25.6 (20.6 ,31.3)	0.0053
Arthritis	35.8 (31.9 ,39.9)	42.6 (37.3 ,48.1)	0.0330
Anemia	3.5 (2.4 ,5.0)	4.9 (3.3 ,7.2)	0.1603
Cancer or malignancy	17.9 (14.7 ,21.6)	20.9 (16.6 ,26.0)	0.3814
Sleep disorder	11.8 (9.3 ,15.0)	8.2 (6.1 ,10.8)	0.1034

Compared to men, women had a significantly higher prevalence of asthma (OR 1.93, 95% CI 1.46 to 2.57, *p* < 0.001) and arthritis (OR 1.77, 95% CI 1.39 to 2.24, *p* < 0.001). Women had a significantly lower prevalence of coronary heart disease (OR 0.48, 95% CI 0.27 to 0.87, *p* = 0.015) and gout (OR 0.42, 95% CI 0.25 to 0.70, *p* = 0.001) after adjusting for age, race/Hispanic status, BMI, smoking status, FEV1% predicted and prevalent comorbidities (including hypertension, congestive heart failure, coronary heart disease, angina pectoris, heart attack, stroke, dyslipidemia, diabetes, gout, asthma, arthritis, anemia, cancer or malignancy and sleep disorders). The adjusted odds ratio for the prevalence of comorbidities between women and men is presented in Table 3.

**Table 3:** Adjusted odds ratio for women compared to men for the prevalence of comorbidities among participants with COPD aged 40–79, U.S. NHANES 2007–2012

Comorbidity	Odds Ratio	Lower 95%	Upper 95%	*P* value
Cardiovascular disease				
Hypertension	1.13	0.88	1.45	0.344
Congestive heart failure	0.73	0. 39	1.36	0.319
Coronary heart disease	0.48	0.27	0.87	0.015
Angina pectoris	0.88	0. 43	1.80	0.735
Heart attack	0.67	0.38	1.16	0.151
Stroke	1.45	0.85	2.46	0.171
Dyslipidemia	0. 91	0.71	1.17	0.465
Diabetes	0.99	0.70	1.39	0.934
Gout	0.42	0.25	0. 70	0.001
Asthma	1.93	1.46	2.57	<0.001
Arthritis	1.77	1.39	2.24	<0.001
Anemia	0.97	0.59	1.60	0.897
Cancer or malignancy	1.21	0.89	1.64	0.223
Sleep disorders	0.74	0.49	1.11	0.141

Adjusted for age, race/Hispanic ethnicity, BMI, smoking status, FEV1% predicted and prevalent comorbidities (including hypertension, congestive heart failure, coronary heart disease, angina pectoris, heart attack, stroke, dyslipidemia, diabetes, gout, asthma, arthritis, anemia, cancer or malignancy and sleep disorders)

### Survival analysis

**Fig. 2:**
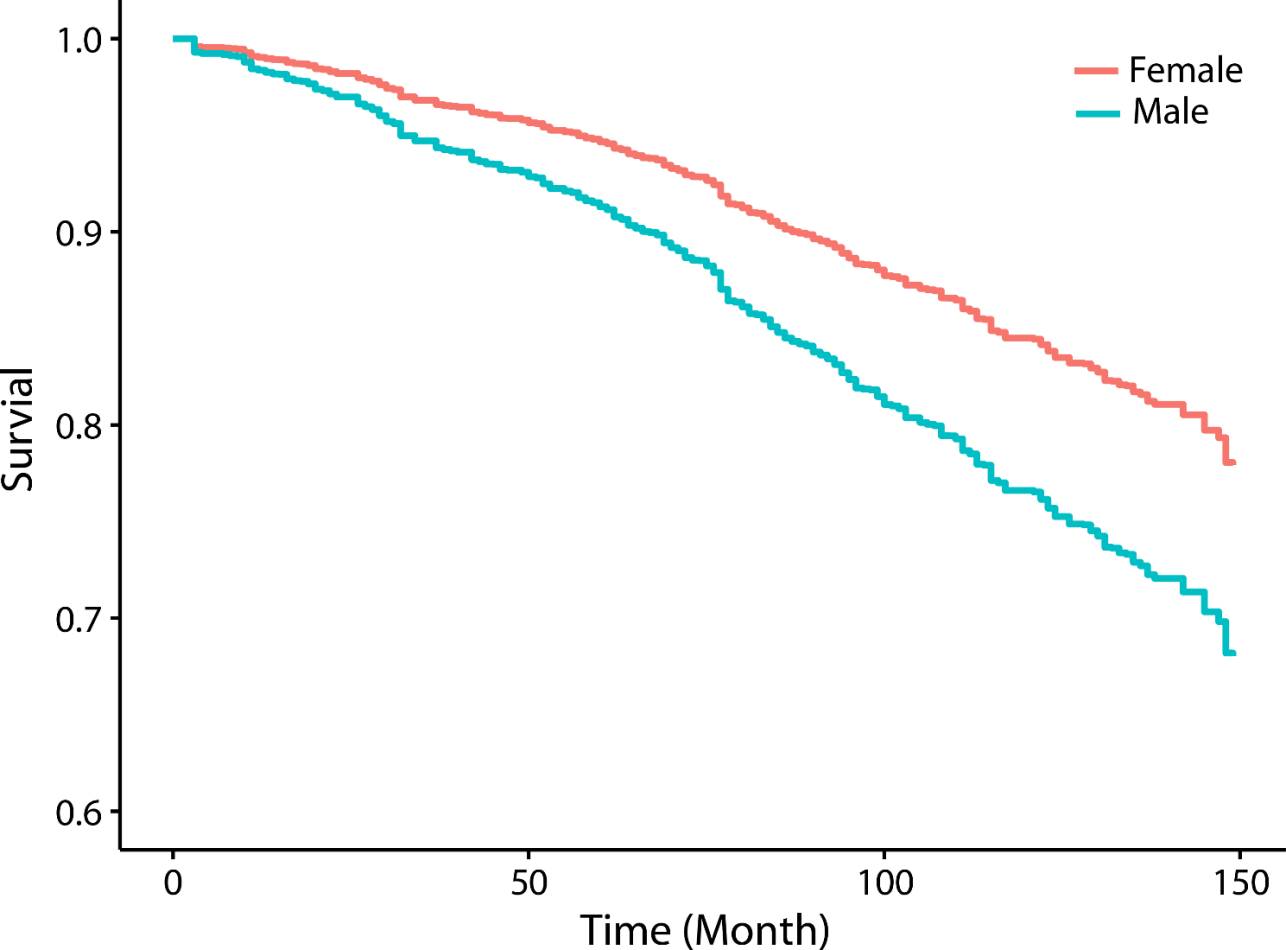
Kaplan–Meier estimates for overall survival among patients with COPD between men and women. Adjusted for age, race/Hispanic ethnicity, BMI, smoking status, FEV1% predicted and prevalent comorbidities (including hypertension, congestive heart failure, coronary heart disease, angina pectoris, heart attack, stroke, dyslipidemia, diabetes, gout, asthma, arthritis, anemia, cancer or malignancy and sleep disorders)

A total of 237 (24.71%) male COPD patients and 105 (18.75%) female COPD patients had died by the end of the follow-up period (by 31 December 2019). Kaplan–Meier analysis found a significant difference in all-cause mortality between the two groups after adjusting for age, race/Hispanic status, BMI, smoking status, FEV1% predicted and prevalent comorbidities (including hypertension, congestive heart failure, coronary heart disease, angina pectoris, heart attack, stroke, dyslipidemia, diabetes, gout, asthma, arthritis, anemia, cancer or malignancy and sleep disorder). Compared with that of the female group, the survival rate of the male group was significantly lower (*p* < 0.001 [log-rank]) (Fig. 2).

For the adjusted survival analysis (Cox’s proportional hazards model), we compared survival between females with COPD and males with COPD, and the crude overall mortality was higher for men than for women (hazard ratio (HR) 1.41 (95% CI, 1.04–1.90; P = 0.025)). This survival benefit for women was not changed in multiple regression models that adjusted for age, race/Hispanic status, BMI, smoking status, FEV1% predicted and prevalent comorbidities (including hypertension, congestive heart failure, coronary heart disease, angina pectoris, heart attack, stroke, dyslipidemia, diabetes, gout, asthma, arthritis, anemia, cancer or malignancy and sleep disorder). The adjusted overall mortality was still higher for men than for women (hazard ratio (HR) 1.63 (95% CI, 1.21–2.20; *P* = 0.001)) (Table 4).

**Table 4:** Association of sex and all-cause mortality among participants with COPD aged 40–79 by sex, U.S. NHANES 2007–2012

	Univariable analysis		Multivariable analysis	
Sex	HR [95% CI]	*P*	HR [95% CI]	*P*
Female	Ref	-	Ref	-
Male	1.41[1.04,1.90]	0.025	1.63[1.21, 2.20]	0.001

Adjusted for age, race/Hispanic, BMI, smoking status, FEV1% predicted and prevalent comorbidities (including hypertension, congestive heart failure, coronary heart disease, angina pectoris, heart attack, stroke, dyslipidemia, diabetes, gout, asthma, arthritis, anemia, cancer or malignancy and sleep disorders)

### Comorbidities on all-cause mortality

**Fig. 3:**
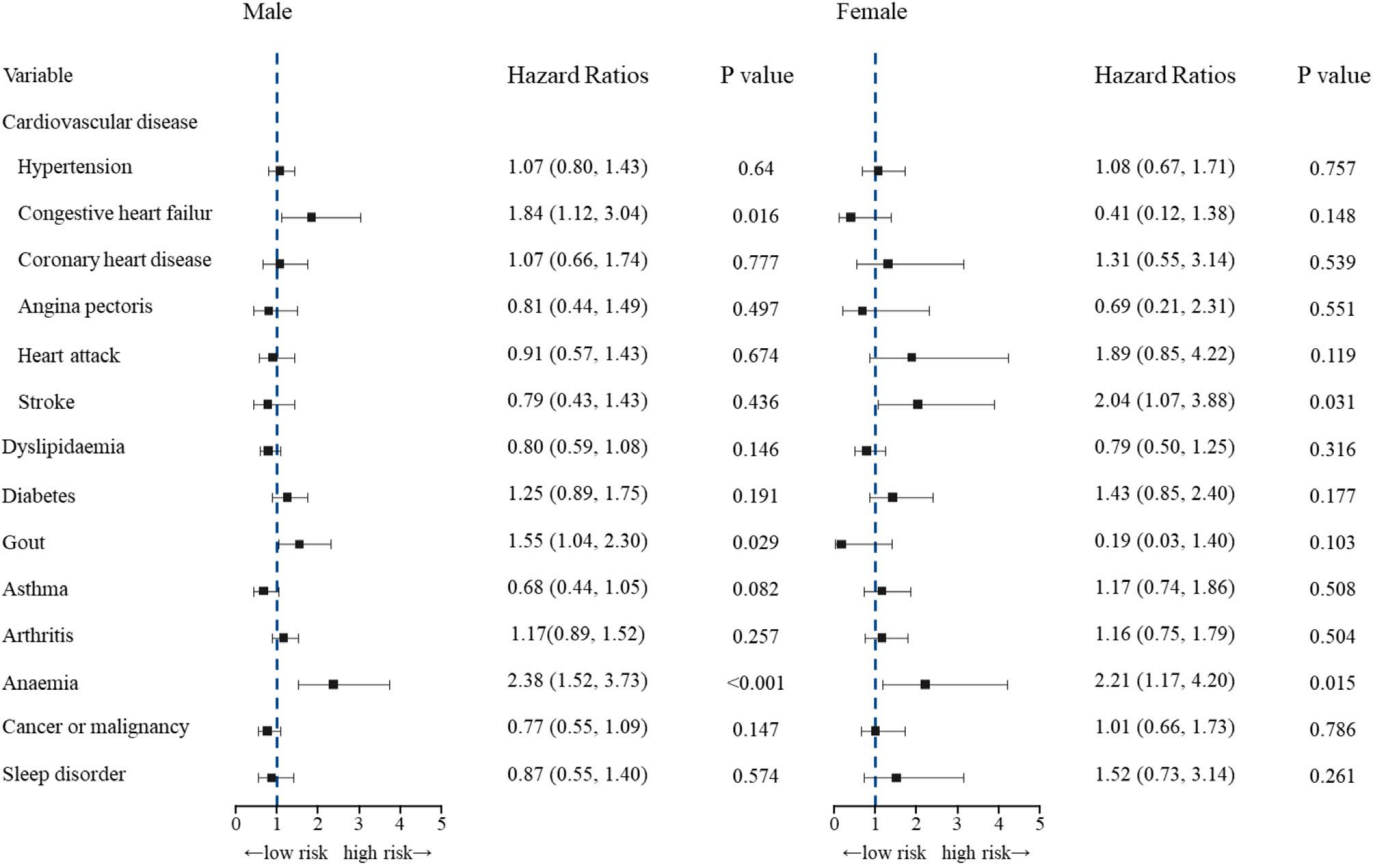
Adjusted Cox regression of the effect sizes of individual comorbidities on all-cause mortality among male and female COPD patients. Adjusted for age, race/Hispanic status, BMI, smoking status, FEV1% predicted and prevalent comorbidities (including hypertension, congestive heart failure, coronary heart disease, angina pectoris, heart attack, stroke, dyslipidemia, diabetes, gout, asthma, arthritis, anemia, cancer or malignancy and sleep disorder)

Among men, among comorbid conditions, notably, the presence of congestive heart failure (HR 1.85, [95% CI 1.12–3.04] *p* = 0.016), anemia (HR 2.38, [95% CI 1.52–3.73], *p* < 0.001) and gout (HR 1.55, [95% CI 1.04–2.30], *p* = 0.029) was related to a significantly higher risk of mortality compared with male COPD patients without these conditions. A history of hypertension, coronary heart disease, angina pectoris, heart attack, stroke, dyslipidemia, diabetes, asthma, arthritis, cancer or malignancy and comorbid sleep disorders were not associated with a higher risk of mortality compared with male COPD patients without these conditions after adjusting for age, race/Hispanic status, BMI, smoking status, FEV1% predicted and prevalent comorbidities (including hypertension, congestive heart failure, coronary heart disease, angina pectoris, heart attack, stroke, dyslipidemia, diabetes, gout, asthma, arthritis, anemia, cancer or malignancy and sleep disorder). Among women, among comorbid conditions, notably, the presence of stroke (HR 2.04, [95% CI 1.07–3.88], *p* = 0.031) and anemia (HR, 2.21, [95% CI 1.17–4.20], *p* = 0.015) was associated with a significantly higher risk of mortality compared with female COPD patients without these conditions. A history of hypertension, congestive heart failure, coronary heart disease, angina pectoris, heart attack, dyslipidemia, diabetes, asthma, arthritis, cancer or malignancy, gout, and sleep disorder were not associated with a higher risk of mortality compared with female COPD patients without these conditions after adjusting for age, race/Hispanic status, BMI, smoking status, FEV1% predicted and prevalent comorbidities (including hypertension, congestive heart failure, coronary heart disease, angina pectoris, heart attack, stroke, dyslipidemia, diabetes, gout, asthma, arthritis, anemia, cancer or malignancy and sleep disorder). Figure 3 shows the adjusted effect sizes for the presence of comorbidities on all-cause mortality among male and female COPD patients.

## Discussion

In this population-based study, the comorbidity distribution characteristics and the impact on mortality exhibited discrepancies between men and women with COPD. Men had a high prevalence of coronary heart disease, heart attack and gout. In contrast, women had a higher prevalence of asthma as well as a high prevalence of arthritis. Male COPD patients with congestive heart failure, anemia and gout had a significantly higher risk of mortality. However, female COPD patients with stroke and anemia had a significantly higher risk of mortality. Anemia had similar effects on mortality between men and women. Compared with the female group, the survival rate of the male group was significantly lower at the end of the follow-up period.

COPD patients often suffer from cardiovascular disease, which affects their functional status and mortality [17]. In our study, men had a pattern of cardiovascular comorbidities characterized by high morbidity with coronary heart disease and heart attack. Compared to men, women had a significantly lower prevalence of coronary heart disease (OR = 0.48, 95% CI 0.27 to 0.87, *p* = 0.015). However, these findings are different from the results of other studies. R. W. Dal Negro et al. showed that congestive heath failure was more prevalent among females [18]. Among cardiometabolic conditions, hypertension, congestive heart failure, angina pectoris, and stroke prevalence did not differ between sexes in the present study, even though a previous report showed a higher morbidity among men [10, 19, 20]. Another study showed that the morbidity of cardiovascular disease was similar between female and male patients with COPD [21]. These results may reflect differences in patient selection and different groups of women and men in the different studies. The oxygen supply to the heart may affect cardiac function, and cardiac disease was more prevalent among men. It is possible that low pulmonary function reduces tissue oxygenation [20]. In addition, certain hormones may influence cardiovascular health in women, such as estrogen [22]. In comparison, male COPD patients with congestive heart failure comorbidities had a significantly increased mortality risk. However, female COPD patients with stroke comorbidities had a significantly increased mortality risk. These notable findings indicate that COPD patients should be actively screened and managed for cardiovascular disease not only because cardiovascular disease differs by sex but also because its impact on all-cause mortality differs by sex.

Interestingly, men had a higher prevalence of gout than women, and gout among male COPD patients was significantly associated with an increased mortality risk, yet gout among female patients was not associated with mortality. K. Kahnert et al. showed that hyperuricemia was associated with an increased mortality risk among patients with COPD [23], but they did not examine men and women separately. The exact reasons for this discrepancy are poorly understood, but sex hormones, especially estrogen, as a uricosuric agent, could play a key role [24].

Anemia has repeatedly been identified as an independent cause of mortality and increased hospitalization rate for COPD patients [2, 25]; it was identified as the most common comorbidity and was associated with a significantly higher risk of mortality among male and female COPD patients in this study. Although anemia clearly appeared to be more frequent in females in absolute terms, there was no significant difference in the distribution of anemia between men and women. Another research group found that anemia was significantly more frequent in females than in males [18]. For the diagnosis of anemia, we used sex-specific hemoglobin cutoff values of less than 12 g/dl for women and less than 13 g/dl for men. Consistent with our findings, another study showed that anemia was independently associated with an increase in premature mortality associated with COPD [24]. Anemia is also a crucial comorbidity linked to older age, nutritional deficiency, cardiovascular disease frequency and poor exercise performance in patients with COPD [2, 7]. One explanation for this finding is a sex-specific concomitant disease interaction of anemia.

Compared to men, women had a significantly higher prevalence of asthma (OR 1.93, 95% CI 1.46 to 2.57, *p*<0.001), and these findings are consistent with those of other studies [26, 27]. A recent systematic review of sex differences in adult asthma and COPD also found that asthma is more prevalent among women [27]. Moreover, the prevalence of asthma was not associated with a higher risk of mortality among men and women in our study. However, we found contradictory results regarding mortality among COPD patients with asthma. A study by Diaz-Guzman et al. [28] showed that COPD patients with asthma had a higher mortality risk during follow-up. On the other hand, another study revealed that concomitant asthma had a protective effect against mortality among COPD patients. Lundbäck et al. [29] showed that COPD patients with concomitant asthma had significantly lower mortality over 20 years of follow-up. These heterogeneous reasons may be due to different patients using different medications, especially inhaled corticosteroids (ICS), which might be relevant in the context of mortality risks among those who have concomitant asthma [30, 31]. Regarding the mortality rate for patients with COPD and asthma, the current evidence is much more heterogeneous. Therefore, this area requires further research.

Few studies have been conducted on sex differences in COPD survival. We found that women had significantly better survival than men after adjusting for differences in baseline confounders. This is consistent with findings from a study by Karin Lisspers et al. [26], which was conducted in Sweden and revealed that the all-cause mortality rate was higher among men than among women (45% vs. 38%). Additionally, J.P. de Torres et al. [32] revealed that all-cause mortality was higher among males than among females (40 versus 18%). However, previous studies have revealed discrepant results regarding the relationship between COPD patient sex and survival. A study by Maeva Zysman et al. [33] revealed no significant difference in survival between men and women matched for FEV1 and age. Similar results were found in the TORCH study; the female and male groups had similar mortality risks once analyses were adjusted for differences in baseline confounders [34]. What is the cause behind this difference? It does not seem to be explained by comorbidity [19]. Our results also indicate that comorbidity does not explain the survival difference between female and male groups. Men have higher mortality rates than women at similar degrees of airflow obstruction, thus explaining why women have lower mortality rates [34]. In addition, men and women may exhibit different COPD phenotypes [19]. Men have higher rates of emphysema than women regardless of the severity of COPD and smoking status [35]. Research shows that emphysema is a strong predictor of mortality [36], which could contribute to the survival difference between men and women with COPD. Another study showed that there was a sex-dependent discrepancy regarding the impact of comorbidities on prognosis among patients with COPD [37]. However, sex differences in COPD survival clearly warrant further study and analysis.

### Strengths and limitations

One of the strengths of this study was that the results are based on data from a large population-based survey obtained from NHANES, which is representative of the total US population, and this study used a survey, follow-up and standardized methods. However, this study has several limitations that must be considered. First, we used a cross*-*sectional study design to address our research aims; thus, direct causal relationships could not be inferred. The clinical or research relevance of these findings needs to be further built upon. Second, there is the possibility of residual confounding, as only selected comorbidities were explored, and we could not evaluate other important diseases recognized as potential comorbidities in COPD patients (for example, obstructive sleep apnea, anxiety, and depression) due to a lack of data in U.S. NHANES. We also were not able to adjust for other (e.g., socioeconomic) factors that may affect men and women unequally and are also associated with mortality. Since we defined all-cause mortality as the primary outcome, data regarding specific causes of mortality were not available, and it was impossible to analyze some specific mortality differences between men and women. Third, postbronchodilator spirometry was not performed for all study participants. This study was limited to the use of prebronchodilator spirometry, which may have misdiagnosed patients without irreversible obstructive lung disease as COPD patients and may have led to an overestimation of the number of COPD subjects. COPD case ascertainment was based on spirometry only (a prebronchodilator FEV1/FVC ratio of less than 0.7); symptoms and/or the presence of known risk factors (e.g., smoking) or a self-report were not considered as part of the COPD case assessment. For numerous reasons, including the fact that other chronic lung diseases can give rise to an obstructive pattern (e.g., bronchiectasis), one would have to question the validity of adopting this approach for COPD case definition. Moreover, meeting the spirometric criterion for COPD is not necessarily synonymous with having the disease, and it is, for example, well known that the fixed ratio criterion will overestimate COPD among elderly individuals. Last, numerous comorbidities in these analyses were collected using self-report questionnaires, and the diagnosis could not be verified from clinical notes; therefore, recall bias is another limitation of this study.

## Conclusions

In conclusion, these data demonstrate that COPD-related comorbidities and all-cause mortality were discrepant between men and women, and men had poorer survival than women in the nationally representative data that were analyzed. These findings suggest that different strategies should be utilized to optimize the management of COPD and its comorbidities between the two sex groups.

## Data Availability

The datasets used and/or analyzed during the current study are available from the corresponding author upon reasonable request.

## Abbreviations

COPD: Chronic obstructive pulmonary disease
NHANES: National Health and Nutrition Examination Survey
DM: Diabetes mellitus
CVD: Cardiovascular disease
FEV1: Forced expiratory volume in one second
FVC: Forced vital capacity
SBP: Systolic blood pressure
DBP: Diastolic blood pressure
BMI: Body mass index
CI: Confidence interval
HR: Hazard ratio
IQR: Interquartile range
SE: Standard error
OR: Odds ratio
